# Multimodal optical coherence tomography and two-photon light sheet fluorescence microscopy for embryo imaging

**DOI:** 10.1117/1.JBO.30.6.060501

**Published:** 2025-06-11

**Authors:** Md Mobarak Karim, Ruijiao Sun, Behzad Khajavi, Manmohan Singh, Yogeshwari S. Ambekar, Alexander W. Schill, Salavat R. Aglyamov, David Mayerich, Kirill V. Larin

**Affiliations:** a University of Houston, Department of Biomedical Engineering, Houston, Texas, United States; b University of Houston, Department of Electrical and Computer Engineering, Houston, Texas, United States; c University of Maryland, Fischell Department of Bioengineering, College Park, Maryland, United States

**Keywords:** optical coherence tomography, one-photon light sheet microscopy, two-photon light sheet fluorescence microscopy, embryonic imaging

## Abstract

**Significance:**

Structural and molecular imaging of the developing embryo can provide deep insights into the development of various pathologies, but few techniques enable the simultaneous detection of these parameters. We demonstrate the first use of combined optical coherence tomography and two-photon light sheet fluorescence microscopy (2P-LSFM) for simultaneous structural and molecular imaging.

**Aim:**

We aim to develop a multimodal high-resolution embryonic system that facilitates simultaneous structural and molecular embryonic imaging.

**Approach:**

We have developed a multimodal imaging system in which the optical coherence tomography (OCT) and light sheet illumination beams were optically co-aligned and scanned through the galvanometer-mounted mirrors and the same illumination objective.

**Results:**

The swept-source OCT system provides a lateral resolution of ∼15  μm and an axial resolution of ∼7  μm. The 2P-LSFM light sheet thickness was ∼10  μm, and the transverse resolution was ∼2  μm. We have demonstrated the system’s capabilities using fluorescent microbeads and fluorescently tagged mouse embryos.

**Conclusions:**

The co-alignment of the OCT and 2P-LSFM systems enables simple image registration and high-throughput multimodal imaging.

## 1 Introduction

Mouse is a widely used mammalian model for studying human disease due to its biological similarities and the accessibility of genetically encoded models. For example, mouse embryonic research has provided profound insights into the genetic and developmental origins of congenital heart disease.^1^^,^^2^ A wide range of imaging methods have been developed for investigating embryonic development, including ultrasound biomicroscopy,^3^ optical projection tomography,^4^ micro-magnetic resonance imaging (micro-MRI),^5^ and micro-computed tomography (micro-CT).^6^ The effectiveness of different imaging methods in capturing this process is influenced by multiple aspects, including sample preparation, environmental conditions, and instrument capabilities. Instrument capabilities include factors such as imaging speed, penetration depth, resolution, contrast, and the amount of energy applied to the sample.^7^ For instance, ultrasound biomicroscopy can achieve ∼50  μm axial resolution using high-frequency ultrasound.^8^ However, this often leads to suboptimal tissue contrast, shallow penetration depth, and artifacts due to changes in backscattering from blood.^3^ Optical projection tomography requires fixed and cleared samples, restricting its use for live imaging.^4^ Micro-MRI also offers a spatial resolution from 25 to 100  μm but requires a long acquisition time (∼2  h) and is therefore not ideal for live imaging.^5^ Micro-computed tomography is capable of high spatial resolution (<100  μm), but the ionizing radiation can be harmful, particularly when conducting longitudinal studies,^6^ and exogenous contrast agents are often required.

Optical coherence tomography (OCT) was originally developed for ophthalmology^9^ but has emerged as a preferred tool in developmental biology.^10^^,^^11^ OCT offers rapid, noninvasive, label-free volumetric imaging without ionizing radiation, the need for exogenous contrast agents or fluorescent targets. In addition, OCT can achieve micrometer-scale spatial resolution and penetrate a few millimeters into highly scattering tissues, making it a preferred tool for imaging live murine embryos during development.^10^ Nevertheless, OCT lacks molecular specificity.

By contrast, several fluorescence imaging techniques have been developed over the last decades for high-resolution molecular imaging with sub-micrometer spatial resolution.^12^ Among them, light sheet fluorescence microscopy (LSFM) has become one of the most popular imaging techniques in developmental biology due to image acquisition speed.^13^^,^^14^ LSFM utilizes a thin sheet of light to illuminate the sample and excite fluorescence, in contrast to slower point-scanning methods such as confocal fluorescence microscopy. Moreover, LSFM can offer a resolution comparable to confocal microscopy at a much faster speed and with reduced photo-bleaching and photo-toxicity.^13^ However, the penetration depth of one-photon LSFM (1P-LSFM) is limited to a few hundred microns in scattering samples such as uncleared and live murine embryos. By implementing two-photon LSFM (2P-LSFM), the penetration depth can be enhanced up to ∼1  mm, depending on sample optical properties.^15^

Although OCT has been combined with two-photon fluorescence lifetime imaging,^16^^,^^17^ it has not been combined with 2P-LSFM, to the best of our knowledge. Building on our previous work where we combined OCT with 1P-LSFM,^18^ we have added 2P-LSFM to significantly enhance the imaging depth in embryonic tissues.

## 2 Methods

### 2.1 Combined OCT and 2P-LSFM System

**Fig. 1:**
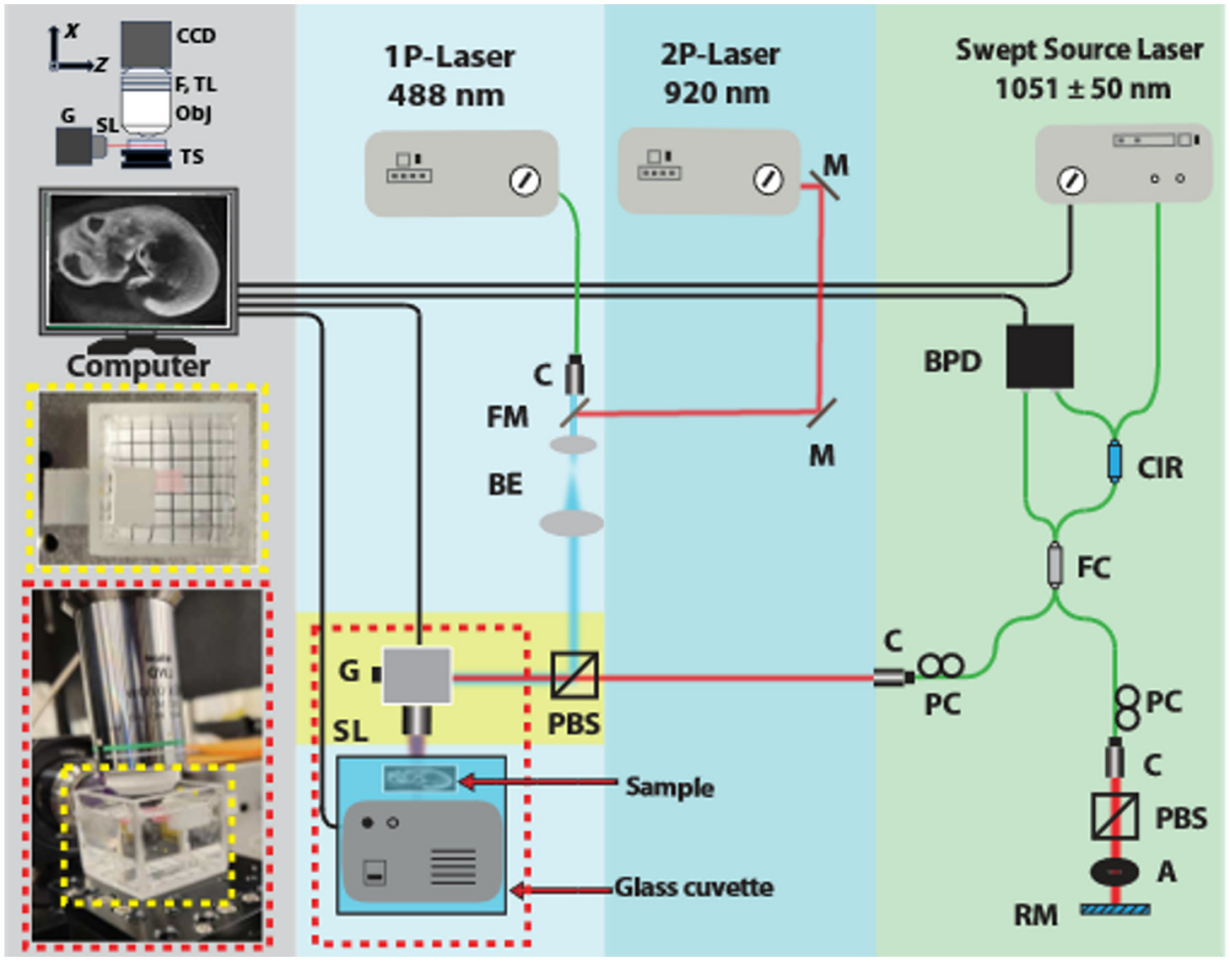
Schematic of the multimodal combined OCT and LSFM system. The gray, light blue, blue, and light green backgrounds correspond to the image collection arm, 1P-LSFM, 2P-LSFM, and OCT, respectively. A: aperture; BPD: balanced photo detector; BE: beam expander; C: collimator; CCD: charged couple device; CIR: circulator; FL: filter; FM: flipped mirror; PBS: polarized beam splitter; FC: fiber coupler; G: galvanometer; M: mirror; PC: polarization control; RM: reference mirror; SL: scan lens; TS: translation stage; TL: tube lens.

Figure 1 shows a schematic for the multimodal OCT and LSFM (OCT-LS) system for imaging mouse embryos. The OCT sub-system is based on a Michelson-style interferometer using a swept source laser (Model 1051 SSOCT, Axsun Tech., Billerica, Massachusetts, United States) with a central wavelength of 1051 nm, bandwidth of 109 nm, and A-line rate of 100 kHz, as shown with a light green background in Fig. 1. Polarization controllers (FPC024, Thorlabs Inc., Newton, New Jersey, United States) in both the sample and reference arms maximized the OCT image signal-to-noise ratio and isolated the OCT light because the systems were combined with a polarization beam splitter (PBS). An identical PBS was used in the reference arm of the OCT system to compensate for the dispersion.

The 2P-LSFM system is based on a femtosecond excitation laser at 920 nm (FemtoFiber Ultra 920, Toptica Photonics Inc., Munich, Germany). The LSFM excitation beam was expanded and directed towards galvanometer-mounted mirrors (GVS002, Thorlabs Inc.) for scanning. In between the beam expander and scanners, the beam passed through the combining PBS, where it was co-aligned with the OCT beam. Notable limitations of traditional LSFM are a short working distance (WD), limited field of view (FOV), and short depth of focus (DoF). To address these limitations, we utilized a telecentric scan lens (LSM03-BB, Thorlabs Inc.) as the illumination objective, which allowed us to maintain long WD, large FOV, and extended DoF. The light sheet (in the Y–Z plane) was generated by scanning one of the two scanning mirrors of the two-axis scanner, whereas the other axis mirror was stepped only for precise focusing. The combined system had a turning mirror to select 1P or 2P excitation pathways. Fluorescence emission was captured using a 16× water immersion objective lens with a numerical aperture (NA) of 0.8 (N16LWD-PF, Nikon Corp., Tokyo, Japan) and a WD of 3 mm. The fluorescence signal passed through the emission objective lens, a filter (520±10  nm), and an infinity-corrected tube lens before reaching the digital camera (C11440-22CU, Hamamatsu, Hamamatsu City, Japan).

The yellow region in Fig. 1 indicates where the OCT and LSFM beams were combined and scanned. Both beams were merged through a PBS that was installed on a tip-tilt stage for precise alignment. For acquiring co-registered images, we consistently maintained the co-planarity of both OCT and LSFM beams. This alignment was verified using a beam viewer camera (LaserCam-HR II 2/3-inch, Coherent Inc., Santa Clara, California, United States). To perform vertical scanning along the axis orthogonal to the image plane, we mounted the sample holder on a motorized translation stage (X-VSR20A, Zaber Tech., Vancouver, Canada). Traditional OCT systems typically use two scanners for 3D imaging, but this would move the light sheet out of the focal plane of the detection arm. Therefore, a motorized stage was used to step the sample incrementally through the LSFM and OCT imaging planes, and OCT and LSFM images were acquired concurrently at each step.

### 2.2 System Characterization

**Fig. 2:**
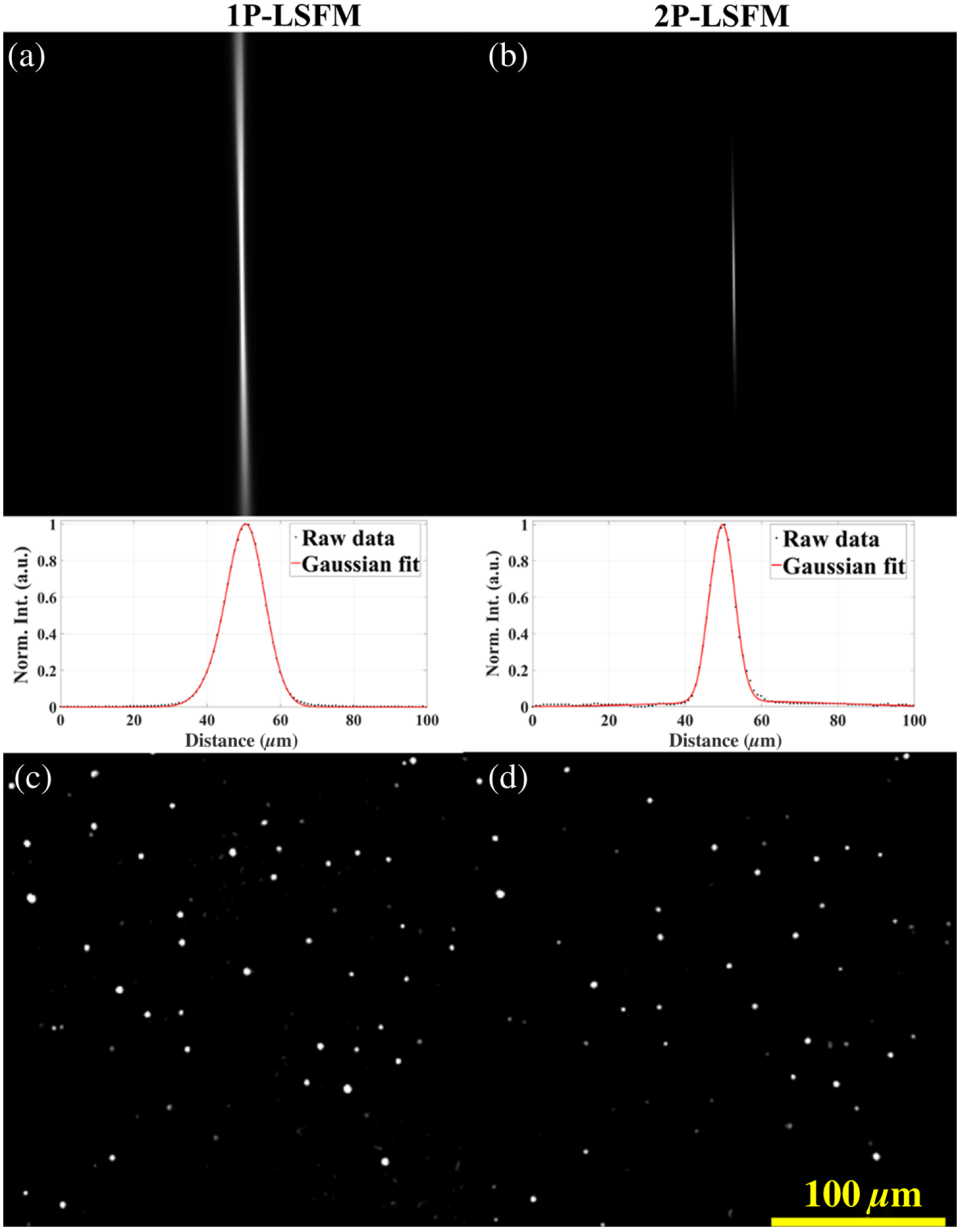
(a) 1P light sheet thickness. (b) 2P light sheet thickness. (c) 1P-LSFM and (d) 2P-LSFM images of 1 to 5  μm diameter microspheres.

Figure 2 visually demonstrates the characterization of the integrated OCT and LSFM system. Initial characterization assessed the transverse (or lateral) resolution of both the OCT and LSFM (both 1P and 2P) systems using a US Air Force resolution target. The OCT lateral resolution was measured by taking an en-face projection of a 3D acquisition of the resolution target, and the resulting OCT lateral resolution was ∼15  μm. We also measured the LSFM lateral resolution with the Air Force resolution target, which was ∼2  μm.

Subsequently, we determined the axial resolution of this integrated OCT and LSFM (1P and 2P) system. As the OCT axial resolution is generally decoupled from the lateral resolution, we measured the axial PSF using a mirror to determine the axial resolution of the OCT sub-system, which was ∼7  μm. The LSFM axial resolution or thickness of the light sheet was measured by determining the beam waist from an image of the stationary beam in a fluorescein solution. Figures 2(a) and 2(b) show the stationary 1P-LSFM and 2P-LSFM beams, respectively. There was no refractive index mismatch because the water immersion detection objective was immersed in the fluorescein solution during the collection of the fluorescence signal. The intensity profile at the beam waist (perpendicular to the illumination direction) was extracted in Fiji^19^ and fit to a Gaussian profile. The FWHM of the 1P beam waist was ∼  11  μm, corresponding to the thickness of the light sheet, i.e., its axial resolution. As expected, the 1P and 2P illumination beams passing through the same objective showed different beam waists, and we observed that the 2P beam was slightly tighter (∼10  μm) at the focal point.^20^ Moreover, both 1P-LSFM and 2P-LSFM show relatively uniform intensity distribution across the light sheet, where the two-photon light sheet was slightly more uniform than the one-photon light sheet due to the nonlinear excitation restricting fluorescence generation to the center of the focal region (Fig. S1 in the Supplementary Material). We ensured precise co-alignment of the light sheet and the focal plane of the detection objective across the entire field of view (FOV) of the LSFM system using a fluorescein solution. Misalignment between the light sheet position and the detector focal plane would reduce the detected fluorescence intensity and blur the image, which would introduce inaccuracies in quantitative measurements.^21^

Finally, we imaged 1 to 5  μm diameter fluorescent microbeads (FMG—Green Fluorescent Polymer Microspheres, Cospheric, Somis, California, United States) to demonstrate the multimodal imaging capabilities of the integrated OCT and LSFM systems. The three-dimensional acquisition involved vertical movement of the sample using a linear stage, which would introduce motion in free-floating beads. To address this issue, the microbeads were embedded in 1% (w/w) low-melting-point agarose (A4718, Sigma-Aldrich Inc., Saint Louis, Missouri, United States). To reduce the refractive index mismatch in the emission path, the agarose-embedded sample was immersed in water. The cuvette and phantom with microbeads were aligned to ensure that the center of the light sheet focused on the focal plane of the detection objective. Figure 2(c) shows one transverse slice of a 3D image from the resulting image stack. The acquisition time for each 1P slice was ∼0.2  s, resulting in a total imaging time of 100 s for the entire stack of 500 images. However, 2P-LSFM imaging was slightly slower (∼0.25  s per slice). The total imaging volume was 1.9×1.9×2.5  mm (x×y×z), with a z-step size of 5  μm for both 1P-LSFM and 2P-LSFM imaging.

### 2.3 Sample Preparation and Imaging

We demonstrate combined structural OCT and molecular LSFM tissue imaging of a mouse embryo at embryonic day (E) 9.5. The murine embryo was stained with acriflavine (3,6-Diamino-10-methylacridinium chloride hydrochloride, Sigma-Aldrich) and embedded in 1% (w/w) low-melting-point agarose. Acriflavine provides a strong contrast for cell nuclei within the surface epithelium, including the absorptive epithelial cells.^22^ The embryo was fixed by immersion in 4% paraformaldehyde (PFA) buffered with 1× phosphate-buffered saline for 2 h at 4°C. The OCT and LSFM images were taken simultaneously from the same region. However, combined OCT and 1P-LSFM, and OCT and 2P-LSFM were acquired separately. The OCT and LSFM data were acquired using a LabVIEW-based (NI, Austin, Texas, United States) interface, and the raw OCT data were processed in MATLAB (MathWorks, Natick, Massachusetts, United States).

## 3 Results

**Fig. 3:**
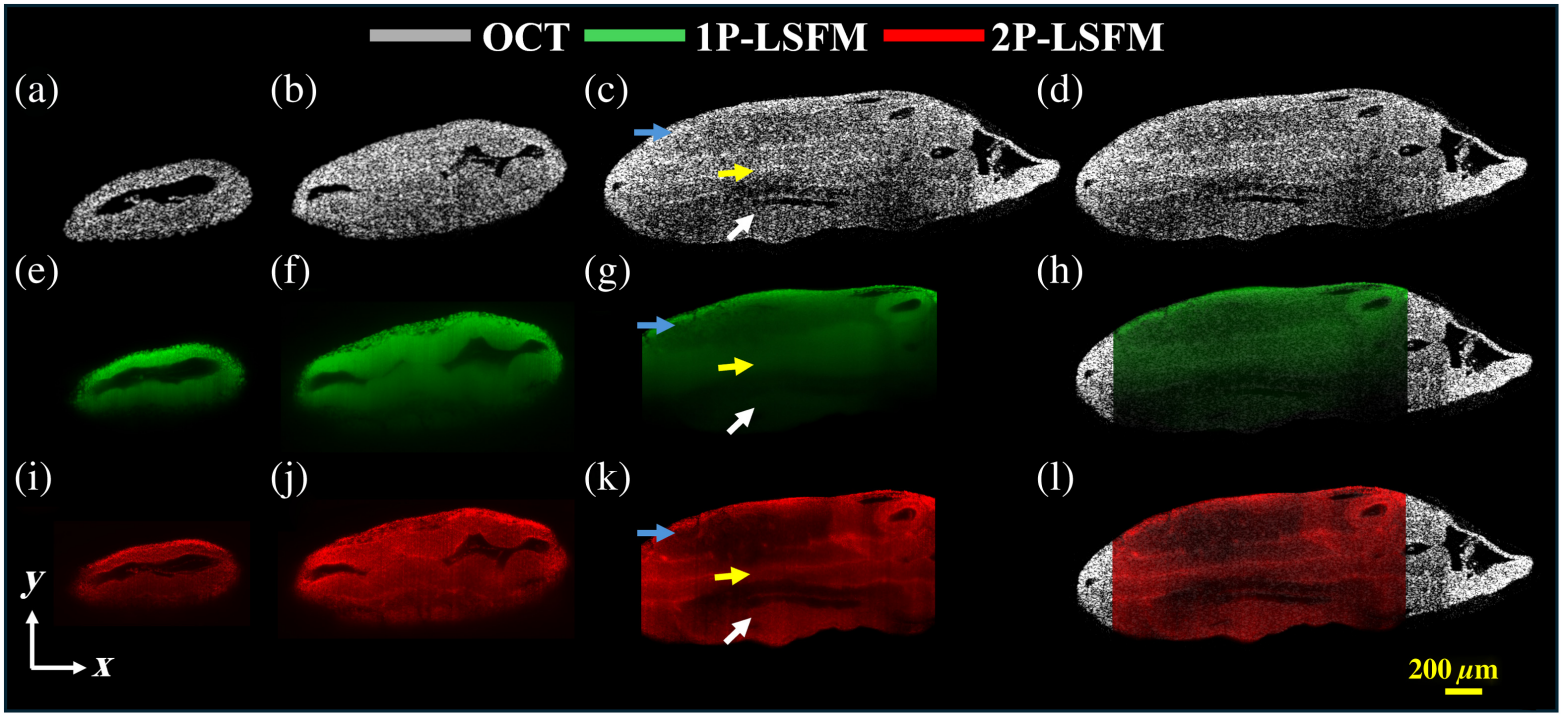
(a)–(c) OCT in gray, (e)–(g) 1P-LSFM in green, and (i)–(k) 2P-LSFM images in red of an acriflavine-stained E9.5 murine embryo taken at (a), (e), (i) 125  μm, (b), (f), (j) 250  μm, and (c), (g), (k) 375  μm below the surface of the embryo. Panels (d), (h), and (l) show OCT, OCT + 1P-LSFM (co-registered), and OCT + 2P-LSFM (co-registered), respectively. The blue, yellow, and white arrows indicate the surface ectoderm, mesoderm, and neuroepithelium layers, respectively.

Figure 3 shows typical OCT and LSFM (1P-LSFM and 2P-LSFM) images. Here, we acquired 500 images with a step size of 5  μm over a 2.5 mm imaging range. Figures 3(a)–3(d), 3(e)–3(g), and 3(i)–3(k) represent OCT, 1P-LSFM, and 2P-LSFM images, respectively, taken at (a, e, i) 125  μm, (b, f, j) 250  μm, and (c, g, k) 375  μm below the embryo surface facing the detection objective. Figures 3(h) and 3(l) show the co-registered images: OCT + 1P-LSFM and OCT + 2P-LSFM, respectively. The incident power of 1P-LSFM was 3 mW, and the camera exposure time was 0.2 s. The 2P-LSFM average power was ∼130  mW with an exposure time of 0.25 s. Despite the utilization of higher power, the femtosecond laser used in 2P-LSFM has low energy deposition per unit time due to the short pulse duration and low duty cycle, which helps to reduce the risk of thermal damage to the sample.^15^ Our imaging protocol is consistent with earlier 2P-LSFM studies that used even higher average powers (up to 200 mW) for long-term imaging of live *Drosophila* embryos without experiencing phototoxic effects.^15^ Using the multimodal system, we were able to perform OCT and LSFM imaging for the same embryo simultaneously. However, the penetration depth of 1P-LSFM was limited to a few hundred microns (∼250  μm) before scattering significantly degraded the resolution. Switching to 2P (920 nm) successfully increased the penetration depth, as seen in Figs. 3(h) and 3(l) with 2P-LSFM superior imaging. Figures 3(i)–3(l) clearly demonstrate the ability of 2P-LSFM to image through the whole embryo coronal plane while preserving resolution at ∼700  μm inside the embryo along the transverse plane, allowing clear visualization of deeper tissue structure. Even though high power can improve the signal-to-noise ratio penetration, the primary cause of superior penetration depth in 2P-LSFM was the longer wavelength and quadratic nature of 2P excitation on photon flux.^15^ The different tissue types can still be distinguished in this coronal plane. For instance, the epithelial tissue along the neural tube is visible due to its higher cellular density in comparison to the surrounding tissue. The penetration depth (∼700  μm of preserved resolution) of 2P-LSFM is clearly superior to 1P-LSFM, which was ∼250  μm for the same sample.

**Fig. 4:**
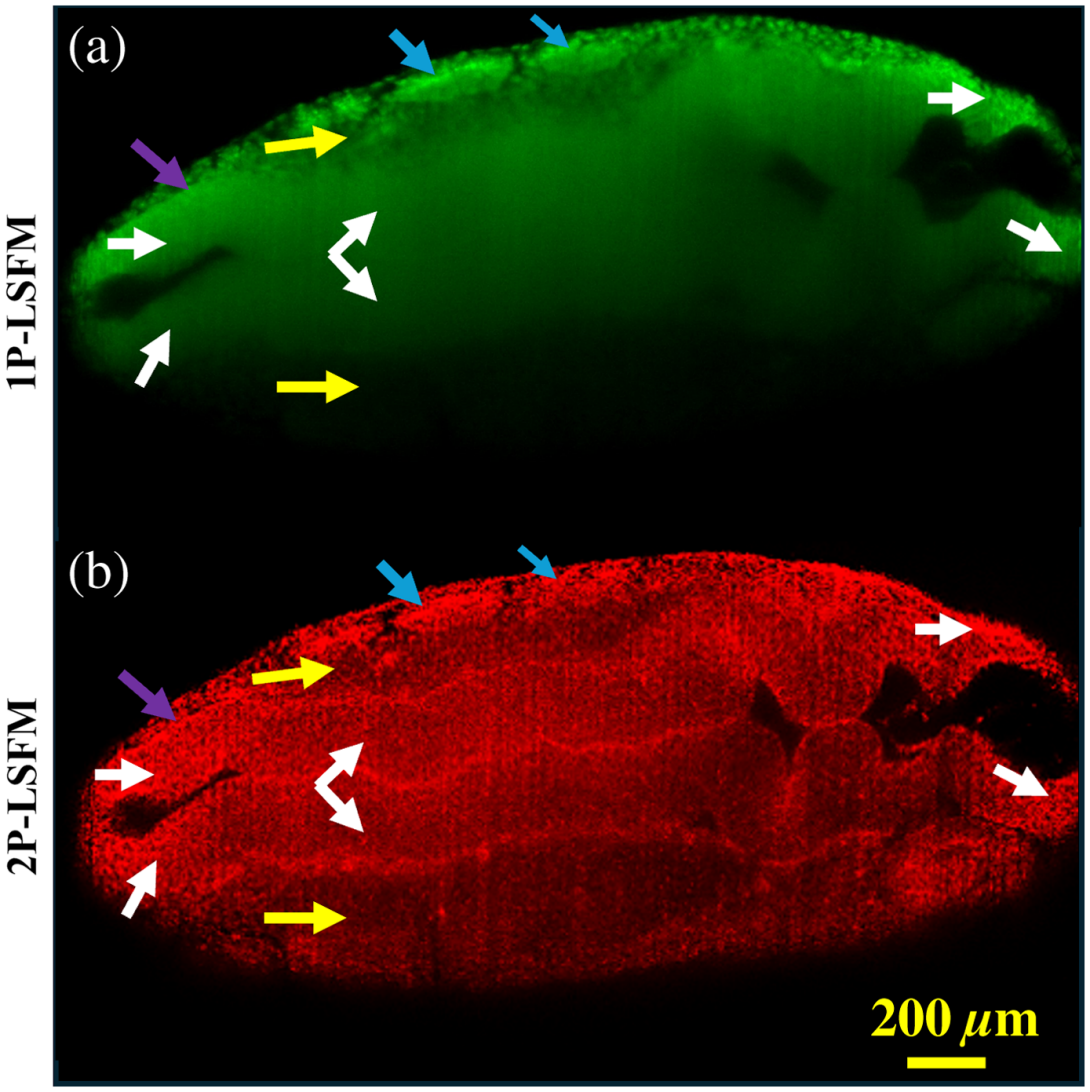
(a) 1P-LSFM and (b) 2P-LSFM images of an acriflavine-stained E9.5 mouse embryo 300  μm below the surface of the embryo. Violet arrows, surface ectoderm; yellow arrows, mesoderm; blue arrows, somites; white arrows, neuroepithelium.

Figure 4 further demonstrates a magnified view of the same coronal plane of the E9.5 acriflavine-stained embryo at 300  μm below its surface as imaged by 1P-LSFM and 2P-LSFM. Figure 4(a) shows that the neural tube tissues cannot be distinguished with 1P-LSFM, and only superficial (relative to the incident beam coming from the top of the image) cells of the mesoderm and surface ectoderm can be distinguished. On the other hand, Fig. 4(b) demonstrates the imaging superiority of 2P inside scattering tissues. The blue, violet, yellow, and white arrows indicate the somites, surface ectoderm, mesoderm, and neuroepithelium, respectively.

## 4 Discussion and Conclusion

Here, we have demonstrated the capabilities of the multimodal OCT and 2P-LSFM system to acquire high-resolution co-registered structural and cellular-level imaging in mouse embryos. We have developed a versatile 1P-LSFM/2P-LSFM/OCT imaging system capable of simultaneous molecular and structural imaging. The incorporation of 2P-LSFM successfully overcame the major limitation of penetration depth of our previously demonstrated 1P-LSFM system,^18^ which was limited to a few hundred microns in scattering tissues. By implementing 2P excitation at 920 nm, we preserved the resolution until   700  μm inside the embryo, enabling imaging deeper inside murine embryos compared with 1P-LSFM, which is crucial for understanding embryonic developmental processes. In particular, the penetration inside the uncleared murine embryo paves the way for imaging dynamic processes in mouse embryos *in vivo*, such as neural tube closure with molecular specificity. Such studies have been accomplished with zebrafish due to their intrinsic transparency,^23^ but murine studies have been limited to fixed and cleared samples.^24^ In addition, image co-registration is trivial because the illumination beams were co-planar, and images were acquired simultaneously.

OCT-LS still has limitations. For the future addition of an incubation chamber for *in vivo* imaging, the WD was relatively long at 25 mm for both OCT and LSFM. The resolution of the LSFM system was, therefore, compromised compared with that of a typical LSFM system that is capable of submicrometer resolution. In addition, the speed of multimodal image acquisition was limited by the exposure time of the LSFM sub-system. The sample was physically stepped using a translation stage to maintain the precise alignment of the LSFM excitation and detection arms. These two parameters resulted in 3D multimodal imaging that was significantly slower than OCT imaging by approximately an order of magnitude. In addition, due to the quadratic dependence of two-photon absorption, the effective excitation volume in 2P-LSFM is more constrained along the light sheet than that in 1P-LSFM. Despite the shorter excitation region, the key benefit of 2P-LSFM is to achieve deeper penetration, particularly in scattering tissue.

The pilot study presented in this work has demonstrated the feasibility of multimodal integrated OCT and 2P-LSFM imaging for studying embryonic development. Future work will focus on implementing an incubator for live imaging, multimodal imaging of embryo cardio-dynamics,^25^ and implementing an actuator for the detection arm to speed up imaging.

## Supplementary Material

10.1117/1.JBO.30.6.060501.s01

## Data Availability

The data that supports the findings of this article can be requested from the authors.
