# Supplementary material for: Multimodal optical coherence tomography and two-photon light sheet fluorescence microscopy for embryo imaging
Source: J Biomed Opt. 2025 Jun 11;30(6):060501. doi: 10.1117/1.JBO.30.6.060501 (PMC12152587; doi:10.1117/1.JBO.30.6.060501)
Supplement: Supplementary file 1 [file JBO_030_060501_SD001.docx]

**Multimodal optical coherence tomography and two-photon light-sheet fluorescence microscopy for embryo imaging**

**Supplementary Fig. 1**


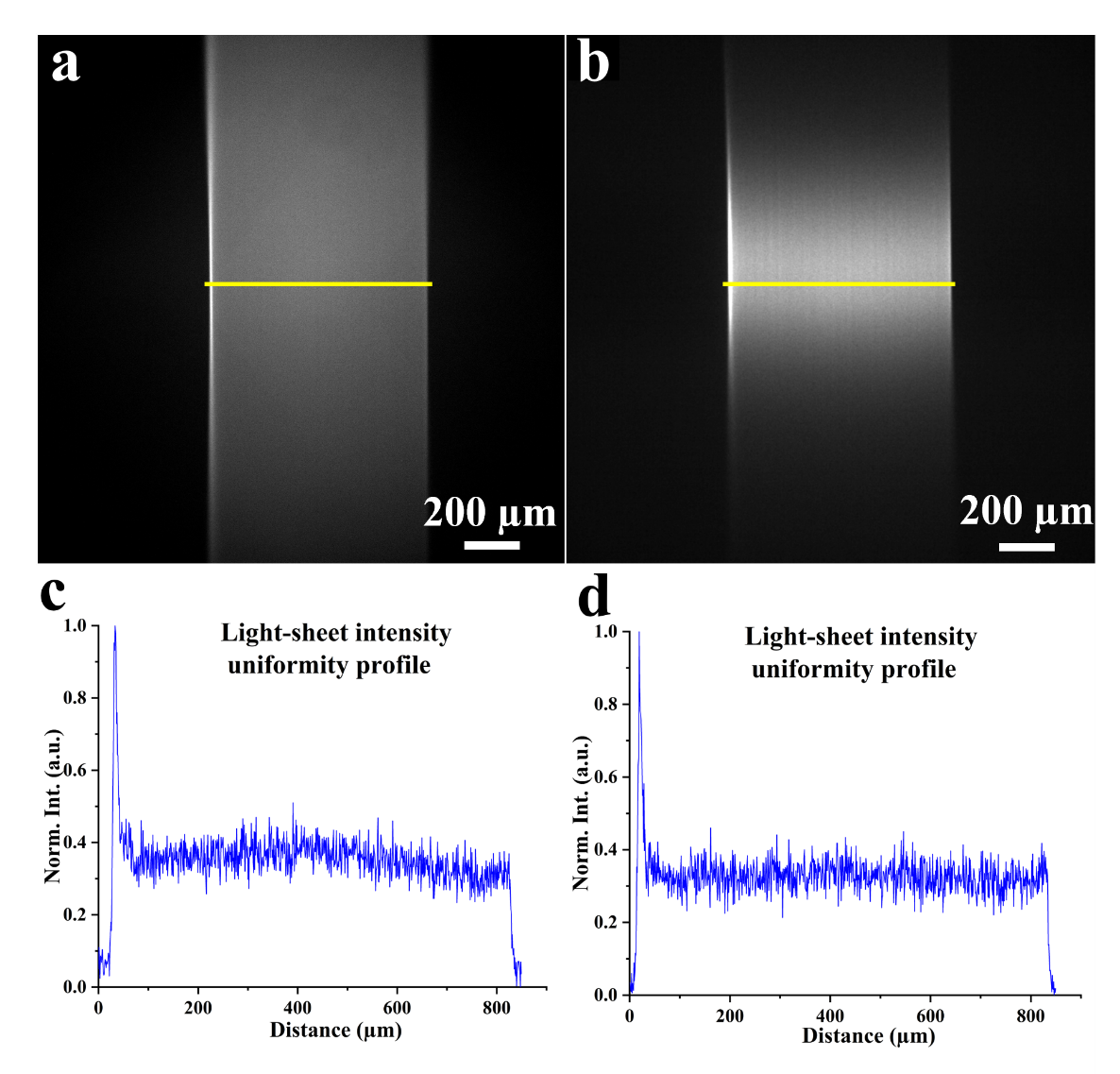


Figure S1. One-photon and two-photon generated light-sheet by the combined OCT-LS system. (a) one-photon LS for ~800 µm scanned area; (b) two-photon LS for ~800 µm scanned area; (c and d) corresponding LS intensity uniformity profile, respectively.

Figure S1 illustrates the beam uniformity profile along the generated LS axis. Both beams have uniform intensity distributions across the LS from the LS intensity profiles shown in Fig. S1(c) and Fig. S1(d). Notably, two-photon LS is slightly more uniform than the one-photon LS due to the nonlinear excitation restricting fluorescence generation to the central or focal region. The presence of the high peak in the intensity profiles is due to the scanner returning to the same position after every scan cycle, which results in localized intensity accumulation.
